# Data for moisture measurements during vertical absorption in building porous materials such as brick and limestone

**DOI:** 10.1016/j.dib.2018.01.067

**Published:** 2018-01-31

**Authors:** Chris Evangelides, George Arampatzis

**Affiliations:** a Department of Hydraulics and Transportation Engineering, Polytechnic School, Aristotle University of Thessaloniki, Thessaloniki 54 124, Greece; b Soil and Water Resources Institute, Hellenic Agricultural Organisation-DEMETER, 57400 Sindos, Greece

## Abstract

This article contains the datasets obtained from experiments in laboratory related to moisture propagation in building porous materials. The datasets contain moisture measurements and corresponding time measurements during vertical infiltration experiment in brick and limestone samples. Moisture measurements were carried out using a γ-ray device and water volume absorption was recorded by a computer controlled digital scale.

**Specifications Table**

**Table:** 

Subject area	*Physics*
More specific subject area	*Moisture profile in porous materials*
Type of data	*Raw and processed data*
How data was acquired	*γ-ray equipment (*^*241*^*Americium 300mci ,scintillation crystal detector Thorn Emi 9266B, Harshaw NA-23,Harsaw NR-30,Harsaw NS-30), Mariotte burette, computer controlled digital scale model Adam PGW 3502e*
Data format	Spreadsheets in MSExcel format *.xls
Experimental factors	*Two samples were examined a limestone sample with 17.2812 cm*^*2*^*base area, 16 cm height and porosity 0.325 cm*^*3*^*cm*^*−3*^*and a brick sample with dimensions 19.935 cm*^*2*^*base area, 13.5 cm height and 0.255 cm*^*3*^*porosity The limestone was from the area of Chania, Crete Greece and the brick was traditional style backed in low temperature (< 1000* *°C). Both samples were wrapped in membrane and the experiment was carried out in constant temperature in order to minimize evaporation.*
Experimental features	*Brick and limestone samples were subjected to infiltration and moisture content in different position and corresponding time was monitored through γ-ray device. Water volume entering and also corresponding time was recorded using a digital scale.*
Data source location	*Laboratory of hydraulics, Rural and surveying engineering, Aristotle University of Thessaloniki, Greece.*
Data accessibility	*Data is included in this article in a separate spreadsheet file.*

**Value of the data**

• Evaluation of theoretical models for moisture profile propagation in porous materials.
• Diffusion coefficient and sorptivity model evaluation.
• Accurate measurements of input water volume since experimental process was not interrupted due to the use of the Mariotte burette.
• Researchers can validate theoretical models with actual data.

## 1 Data

Data is given in spreadsheet form as a separate file.

The first spreadsheet contains moisture content measurements in two positions and the corresponding time for limestone. This spreadsheet is named “*θ*(*t*) limestone” and the two positions were 3 and 8.5 cm from bottom, where moisture measurements were obtained.

The second spreadsheet contains cumulative intake water records versus time for the same sample and is named “digital scale limestone”.

The third spreadsheet contains moisture content measurements in two positions and the corresponding time for brick. This spreadsheet is named “*θ*(*t*) brick” and the two positions were 3 and 9 cm from bottom, where moisture measurements were obtained.

The fourth spreadsheet contains cumulative intake water records versus time for the same sample and is named “digital scale brick”.

## 2 Experimental design, materials and methods

Moisture content measurements were carried out using a γ- ray device. The bulk densities and the moisture content were measured by γ-ray absorption method [1], [2], [3]. The device of γ-ray contained a ^241^Am 300 mCi source. The Americium source and the photomultiplier detector (including a NaI crystal and preamplifier) were set on a platform connected to a stepper motor for vertical movement. In this way, the development of water profiles over time was monitored.

The γ-ray method is a laboratory application of a physical phenomenon and specifically the property of a material to absorb part of the incident rays. The percentage of the rays that are bounded depends on many factors but mainly on the density of the material. The isotope ^241^Am was identified by Seaborg, James, Morgan and Ghiorso late in 1944 at the wartime Metallurgical laboratory of the university of Chicago as a result of successive neutron capture reactions by plutonium isotopes in a nuclear reactor [4].

Americium has nuclei that without any external excitation transmit a wide range of radiations. One of the radiations transmitted is γ-ray that is high energy photons. γ-rays are composed from high energy photons. During the transition of nuclei from an energy level to lower one, a photon with energy equal the difference of the energies is produced. Photons ionize the material that they penetrate. This phenomenon is the operation principal of most scintillation detectors. Finally, the γ radiation passing through a material supplies useful information. When a porous media is absorbing water, the water is eventually replacing the air in all pores till saturation. The absorption coefficient for water and air under normal laboratory conditions are almost the same, but the density of the water is much higher that the corresponding one for air [5]. Based on the above simplifications, the equation, giving the intensity of the γ-ray radiation passing through a wetted porous media, is:

(1) $$\frac{{Ι}_{d}}{I_{w}}=\;exp\;(-\;\chi \;\;\theta \;\;\mu_{w}\;\;\rho_{w})$$

where *I*_d_ is the intensity of the rays after passing through a dry porous media, *I*_w_ is the intensity of the rays after passing through a wetted porous media, *χ* the thickness of the media, *θ* is the moisture content of the media, *μ*_w_ is the water absorption coefficient and *ρ*_w_ is the water density. Solving the Eq. (1) for the moisture content of the media:

(2) $$\theta =\;\;\left(\frac{1}{\chi \;\;\mu_{w}\;\rho_{w}}\right)\;\;\;\;ln\;\left(\frac{I_{d}}{I_{w}}\right)$$

Since the number of counts of the scintillation counter in a specific time frame is proportional to the intensity of the radiation:

(3) $$\frac{I_{d}}{I_{w}}=\frac{N_{d}}{N_{w}}$$

where *N*_d_ is the number of counts after passing through a dry porous media, *N*_w_ is the number of counts after passing through a wetted porous media.

So Eq. (2) becomes:

(4) $$\theta =\;\;\left(\frac{1}{\chi \;\;\mu_{w}\;\rho_{w}}\right)\;\;\;\;ln\;\left(\frac{N_{d}}{N_{w}}\right)$$

Eq. (4) gives the moisture content of the media in any position given that *N*_d_ is known at the same position.

The experiment was carried out in the laboratory for two types of building materials in order to measure the moisture content and the inlet water quantity during infiltration [6].

The two samples were a limestone with 17.2812 cm^2^ base area, 16 cm height and porosity 0.325 cm^3^ cm^−^^3^ and a brick sample with dimensions 19.935 cm^2^ base area, 13.5 cm height and 0.255 cm^−3^ porosity. The limestone was from the area of Chania, Crete, Greece and the brick was traditional style backed in low temperature (<1000°C). 1000 °C). Both samples were wrapped in membrane and the experiment was carried out in constant temperature in order to minimize evaporation.

**Fig. 1:**
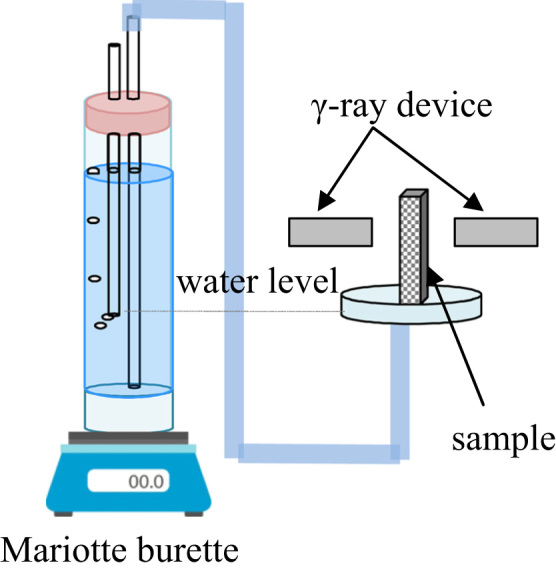
Experimental set up.

The samples were positioned on a pot for wetting and fixed in a specific position so that the γ-rays were aiming at the center and perpendicular through out the whole vertical length of the sample. Next, γ- ray measurements were obtained at specific distances from the base of the sample, while the samples were dry. Constant head was maintained at about 2 mm through the use of a Mariotte burette, which was positioned on a digital scale (Fig. 1). Water inlet volume was automatically recorded per minute for the duration of the experiment. Water inlet quantities versus time and γ-ray counts at specific time steps were recorded at the same positions that dry measurements took place. Moisture content θ was calculated from these counts utilizing Eq. (4) at specific positions. The whole experiment was designed such as that the accuracy of the resulting profiles could be verified from the calculated sorptivity through infiltration and corresponding time measurements.
